# Gut microbiota decreased inflammation induced by chronic unpredictable mild stress through affecting NLRP3 inflammasome

**DOI:** 10.3389/fcimb.2023.1189008

**Published:** 2023-05-24

**Authors:** Li Huang, Zewei Ma, Xiaolei Ze, Xinrui Zhao, Meilin Zhang, Xia Lv, Yunqin Zheng, Huan Liu

**Affiliations:** ^1^ Department of Nutrition and Food Science, School of Public Health, Tianjin Medical University, Tianjin, China; ^2^ Tianjin Key Laboratory of Environment, Nutrition, and Public Health, Center for International Collaborative Research on Environment, Nutrition and Public Health, Tianjin, China; ^3^ BYHEALTH Institute of Nutrition & Health, Science City, Guangzhou, China

**Keywords:** microbiota, probiotic, chronic unpredictable mild stress, NLRP3 inflammasome, fecal transplantation

## Abstract

Dysbiosis of the gut microbiota is associated with the development of depression, but the underlying mechanism remains unclear. The aim of this study was to determine the relationship between microbiota and NLRP3 inflammasome induced by chronic unpredictable mild stress (CUMS). Fecal transplantation (FMT) experiment was conducted to elucidate the potential mechanism. Levels of NLRP3 inflammasome, microbiota, inflammatory factors and tight junction proteins were measured. CUMS stimulation significantly increased the levels of NLRP3, Caspase-1 and ASC in brain and colon(*p*<0.05), decreased the levels of tight junction proteins Occludin and ZO-1 (*p*<0.05). Interestingly, increased NLRP3 inflammasome and inflammatory cytokines and decreased tight junction proteins were found in antibiotic-treated (Abx) rats received CUMS rat fecal microbiota transplantation. Furthermore, fecal microbiota transplantation altered the microbiota in Abx rats, which partially overlapped with that of the donor rats. Importantly, probiotic administration amended the alteration of microbiota induced by CUMS treatment, then reduced the levels of NLRP3 inflammasome and inflammatory factors. In conclusion, these findings suggested that depression-like behaviors induced by CUMS stimulation were related to altered gut microbiota, broke the intestinal barrier, promoted the expression of NLRP3 inflammasome and elevated inflammation. Therefore, improving the composition of microbiota *via* probiotic can attenuate inflammation by amending the microbiota and suppressing the activation of NLRP3 inflammasome, which is considered as a novel therapeutic strategy for depression.

## 1 Introduction

Recently, increasing studies have indicated that inflammation is one of the pathogeneses of depression (Kaufmann et al., 2017). Increased levels of interleukin (IL)-1β, tumor necrosis factor-α (TNF-α) and interleukin (IL)-18 were found in depression patients and animals (Perry et al., 2021; Yang et al., 2021), and depressive-like behaviors were ameliorated by anti-inflammatory approaches (Köhler et al., 2014). When the inflammasome was activated, the level of inflammation was increased and resulted in the development of inflammatory diseases, including depression (Guo et al., 2015).

NLRP3 inflammasome is a crucial inflammasome that consists of, including nod-like receptor protein 3, adaptor protein ASC and procaspase-1 precursor (Haneklaus et al., 2013). Activated NLRP3 inflammasome by diverse factors, such as bacteria, fungi, endogenous danger associated molecular patterns (DAMPS) including mitochondrial DNA, Adenosine triphosphate (ATP) and reactive oxygen species (ROS) could promote the maturation of caspase-1, leading to the production and release of proinflammatory cytokines such as IL-1β and IL-18 (Heneka et al., 2018). Growing evidence suggested that NLRP3 inflammasome was associated with depression, Alzheimer’s diseases and other diseases. Increased levels of NLRP3, caspase-1 and ASC in peripheral blood mononuclear cells or plasma have been found in depression patients (Alcocer-Gómez et al., 2014; Syed et al., 2018). Similar results have also been demonstrated in animal studies (Arioz et al., 2019; Wang et al., 2021). What’s more, depression-like behaviors could be improved by knocking or suppressing the NLRP3 gene (Su et al., 2017; Li et al., 2021). Furthermore, the activation of NLRP3 inflammasome in prefrontal cortex in depression rats can be suppressed by fluoxetine, which is a widely used antidepressant (Pan et al., 2014). Given that NLRP3 inflammasome may be play a key role in depression.

Microbiota, as an environmental factor, can impact mood through the microbiota-gut-brain axis (Sarkar et al., 2016). Both clinical and animal studies found that altered microbiota was related to depression (Desbonnet et al., 2015; Jiang et al., 2015), and rats subjected to antibiotics treatment also replicated depressive behaviors when transplanted with the microbiota from depressive patients or animal (Zheng et al., 2016; Lai et al., 2021). However, the detailed mechanisms regarding the effects of microbiota on depression have not been determined. Currently, studies indicated that NLRP3 inflammasome may be a bridge between stress and stable intestinal environment, suggesting that the effects of microbiota on depression may be associated with the NLRP3 inflammasome (Hao et al., 2021). When microbiota was cleared by broad spectrum antibiotics, the NLRP3 inflammasome in the hippocampus was activated, which promote production and release inflammatory factors, leading to elevated levels of inflammation in brain (Lowe et al., 2018). On the other hand, NLRP3 inflammasome also can affect the abundance of microbiota to influence some disease, for example pancreatitis (Fu et al., 2018), colitis (Zhen and Zhang, 2019), depression-like behaviors (Zhang et al., 2019). Meanwhile, inhibition of caspase-1 levels through knockout gene or inhibitor can modulate the gut microbiota composition, thus alleviating depression-like behaviors (Wong et al., 2016). These evidences supported the concept of a gut-microbiota-inflammasome-brain axis, which suggested that microbiota can affect depression *via* inflammasome (Miao et al., 2011). In addition, microbiota could damage gut barrier function through decreasing Occludin and ZO-1 levels, contributing to the release of pro-inflammatory substances into the circulation and the increase of systemic inflammation and oxidative stress, which was relevant to the etiology, progression, and treatment of many neuropsychiatric disorders (Julio-Pieper et al., 2014; Anderson et al., 2016).

Our previous studies have found that chronic unpredictable mild stress (CUMS) stimuli induced depression-like behaviors, increased levels of IL-1β and IL-18 and altered the microbiota (Huang et al., 2022), which suggested NLRP3 inflammasome might be activated. What’s more, intervention with combined probiotic reversed altered microbiota and reduced levels of inflammatory cytokines, further indicating that microbiota might be a key role in activating NLRP3 inflammasome and upregulating inflammation (Mou et al., 2022). Thus, the aim of this study was further explored the effects of microbiota on NLRP3 inflammasome and the possible mechanism of probiotic antidepressant. In order to test this hypothesis, two experiments were performed. We used CUMS model of depression and added probiotic administration as a comparison to explore the role of microbiota on NLRP3 inflammasome. Subsequently, the fecal transplantation (FMT) experiment, a microbiota-targeted technique, was performed to further identify the effect of microbiota on NLRP3 inflammasome.

## 2 Materials and methods

### 2.1 Experimental animal

Male Sprague-Dawley (SD) rats were purchased from SPF Biotechnology Co,Ltd (Beijing,China) for this study. At the beginning of the study, the weight of each rat is 180-200g. All rats were kept under a 12h light/dark cycle at a constant temperature and humidity with ad libitum access to food and water. Rats were allowed to acclimate to housing conditions for a week prior to further treatments. All animal experiments were approved by the Animal Ethical and Welfare Committee of Tianjin Nankai Hospital (NKYY-DWLL-2020-180) and complied with the national and international guidelines for the Care and Use of Laboratory Animals.

### 2.2 Experimental design

#### 2.2.1 Stage 1

**Figure 1:**
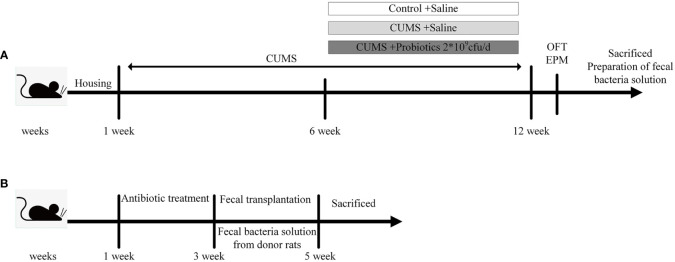
Experimental design. **(A)** The experimental design of stage 1. **(B)** The experimental design of stage 2. CUMS, chronic unpredictable mild stress; OFT, open field test; EPM, elevated plus maze.

In our previous study (Huang et al., 2022), rats that experienced CUMS stimulation were administered with Lactobacillus rhamnosus HN001 (HN001) and/or Bifidobacterium animalis subsp. lactis HN019 (HN019) to explore the effect of probiotic on depression-like behaviors. We demonstrated that CUMS exposure increased IL-1β and IL-18 levels, which are generated and released by an active NLRP3 inflammasome. However, our intervention with probiotics, either alone or in combination, led to a reduction in inflammatory factor levels and an improvement in depression-like behavior. Interestingly, the combination probiotic intervention outperformed a single probiotic in terms of enhancing microbiota and reducing IL-1β and IL-18 levels. We carried out a study using three donor groups—a Control group, a CUMS group and a combination probiotic—to further illustrate the possible connection between gut microbiota and NLRP3 inflammasome. Twenty-four male rats were randomly allocated into three groups (8 rats per group), including Control (Control group not subjected to any stress), CUMS (CUMS group subjected to CUMS procedure), Probiotic (probiotics treatment and CUMS procedure). After 6 weeks CUMS stimulus, the control group and CUMS group were given gavage saline 1mL/day, the probiotic group were given gavage probiotic 2*10^9^cfu/day. The stressors and CUMS procedure were performed as previously described (Huang et al., 2022). All rats which were euthanized with CO_2_ after fasting overnight were sacrificed after completion of behavioral tests. Brain and colon tissue either fixed with 4% (w/v) paraformaldehyde for immunohistochemical staining or stored at −80°C after liquid nitrogen flash-freezing for biochemical tests. According to the other studies (Li et al., 2019; Yan et al., 2020), the cecum content was collected and mixed from the same group, then immediately diluted 40-fold in PBS. After centrifugation at 100×g for 5 min at 4°C, the supernatant was collected under sterile conditions and stored at –80°C until fecal transplant (FMT) (**Figure 1A**).

#### 2.2.2 Stage 2

In the second stage of experiment, 24 male Sprague-Dawley (SD) rats (8 weeks old) were randomly divided into 3 groups (8 rats per group): FMT-Control, FMT-CUMS, FMT-Probiotic. All rats were orally administered an antibiotic cocktail of ampicillin, kanamycin, metronidazole, neomycin (all at 0.25 mg/day), and vancomycin (0.125 mg/day) for 14 consecutive days to deplete the microbiota to build the antibiotic-mediated microbiota depletion rat model. Then antibiotic-treated rats were orally administered 200 μL fecal supernatant from the donor rats of experiment 1 for the next 2 weeks (Yan et al., 2020). Finally, rats were euthanized under CO_2_ anesthesia, and their cecum content, brain and colon were collected and immediately stored at −80°C for further detection (**Figure 1B**).

### 2.3 Interleukin-1β and interleukin-18 detection

IL-1β and IL-18 levels in brain and colon were measured using rat enzyme-linked immunosorbent assay (ELISA) kits, according to the manufacturer’s instructions (MM-0047R1 and MM-0194R1, Meimian industrial Co, Ltd, Jiangsu, China). The brain and colon were homogenized in an ice bath and then centrifuged at 15000 rpm for 15 min. The supernatants were collected and immediately used to measure the concentration of inflammatory factors. The total protein concentrations of supernatants were determined with Enhanced BCA Protein Assay Kit (SparkJade, Shandong, China). The final results of IL-1β and IL-18 levels in brain and colon were normalized to the total protein concentration of each brain and colon supernatant accordingly.

### 2.4 Western blot analysis

Dissected whole brain and colon tissues were homogenized and lysed in RIPA buffer (SparkJade, China), incubated on ice for 30 min, and centrifuged at 14,000 g for 20 min at 4 °C. Then, the supernatant was collected using centrifugation and the total protein concentration was determined in the cleared lysates with the BCA protein assay kit (SparkJade, China). Equal amounts of protein from each sample were separated by 12% sodium dodecyl sulfate-polyacrylamide gel electrophoresis and transferred to PVDF membranes (Millipore, Schwalbach, Germany) by wet electrical transfer method. Subsequently, the membranes were blocked with 5% milk in 1× Tris buffered saline Tween for 1 h at room temperature. After blocking, the membranes were incubated overnight with antibodies against, NLRP3 (1:2000, Bioss), caspase-1 (1:2000, Bioss), ASC (1:2000, Bioss), Occludin (1:2000, Bioss), ZO-1 (1:2000, Bioss). Then, secondary antibody (horseradish peroxidase-linked anti-rabbit IgG, 1:10000; all from SparkJade, China) were incubated for 1 h at room temperature. The blots were developed by immobilon western chemiluminescent horseradish peroxidase substrate and observed using a ChemiDocTM XRS Imaging System (Bio-Rad, Hercules, CA, USA). The band levels were quantified using ImageJ software (National Institutes of Health, Bethesda, MD, USA). To control sampling errors, the ratio of band intensities to GAPDH was obtained to quantify the relative protein expression level.

### 2.5 Immunofluorescence staining

The brain was removed and postfixed in 4% paraformaldehyde at 4°C overnight and then embedded in paraffin. All wax blocks were sectioned to 5-μm thickness by a fully automatic slicer, followed by conventional dewaxing to hydration. The sections were then treated with 3% H_2_O_2_ for 10 min and rinsed thoroughly in distilled water, washed in PBS buffer for 5 min. Subsequently the tissues underwent antigen retrieval by incubating the sections for 6 min in a solution of citric acid at 90°C. The brain sections were blocked with goat serum for 1 h at 37°C. Then, the primary antibodies against NLRP3 (1:100), caspase-1 (1:100), and ASC (1:100) were added. Then incubated with TRITC-conjugated goat anti-rabbit secondary antibodies (1:100) (zhongshanjinqiao, Bejing, China) for 1 h at 25 °C. The nuclei were stained with 4′,6-diamidino-2-phenyldiazole, dihydrochloride (DAPI) (10 μg/mL, SparkJade, China) 10 min before mounting. The fluorescence images were obtained with an inverted microscope (IX81; Olympus, Tokyo, Japan). The ratio of the number of red (positive cell) and blue (DAPI) were counted and quantified by Image Pro Plus 6.0 software (Media Cybernetics, Silver Spring, MD, USA).

### 2.6 16S rRNA sequencing

The experiments included extraction of total DNA from cecum content samples and analysis of microbial composition *via* 16S rRNA sequencing. The detailed experimental method has been described in our previous study (Huang et al., 2022).

### 2.7 Statistical analyses

The data were analyzed using IBM SPSS Statistics 24.0 software (IBM Corp, USA) and are expressed as the mean ± standard deviation (SD). After verifying normal distribution of data, comparisons among different groups were performed by two-way ANOVA with LSD t test for comparison between every two groups. P values of 0.05 or less were considered significant. GraphPad Prism 5 (GraphPad Software, La Jolla, CA) was used to generate graph. To confirm differences in the abundances of individual taxonomy between the two groups, STAMP software was utilized. LEfSe was used for the quantitative analysis of biomarkers within different groups. We rarified the OTU table and calculate four metrics: Chao1 index, ACE index, Simpson index and Shannon index to compute Alpha diversity. PCoA based on weighted and unweighted UniFrac metrics was used to assess the variation of bacterial composition among different groups. To identify differences of microbial communities between the different groups, ANOSIM and ADONIS were performed based on the Bray-Curtis dissimilarity distance matrices. The relative abundance of fecal microbiota in the three groups was compared using the Kruskal–Wallis H test. A value of *p*<0.05 was considered statistically significant.

## 3 Results

### 3.1 CUMS increased the expression of NLRP3 inflammasome

**Figure 2:**
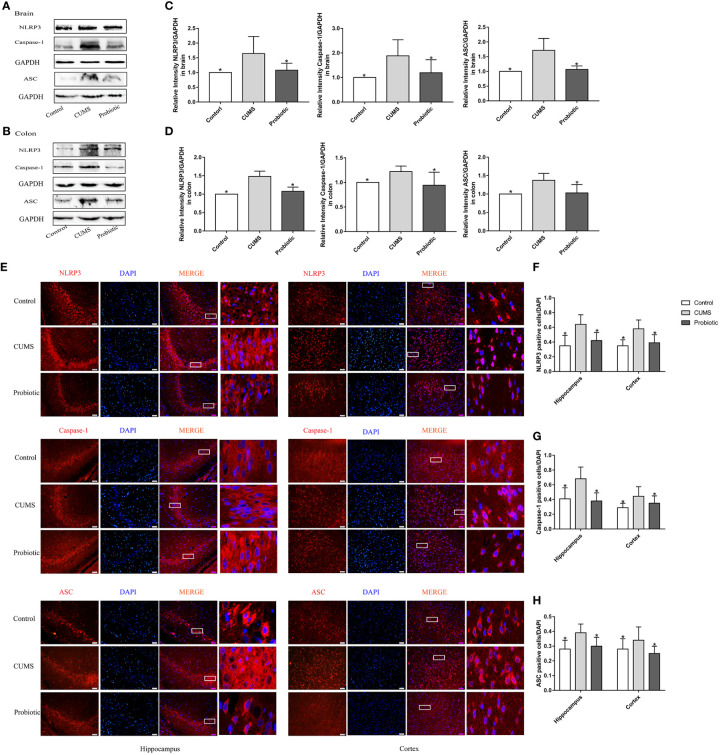
CUMS increased the expression of NLRP3 inflammasome in brain and colon, but probiotic alleviated the upregulation. The expression of NLRP3 inflammasome in brain **(A)** and colon **(B)** were detected by western blot. **(C, D)** Quantitative analysis for NLRP3 inflammasome in brain and colon. **(E)** Immunofluorescence analysis of NLRP3 inflammasome in hippocampus and cortex. Representative images of NLRP3 inflammasome (red) staining in CA3 area of the hippocampus and cortex. Nuclei were stained with 4,6-diaminido-2-phenylindole (DAPI, blue). Merge image shown the positive cells expressed NLRP3 inflammasome. Each right-hand column depicts a magnified image of the rectangular region of the corresponding image in the left column. Scale bar=50 μm. **(F-H)** Quantification of NLRP3 positive cells in the hippocampus and cortex. All data are expressed as the mean ± SD (n=5 in western blot, n=3 in immunofluorescence). *p<0.05, compared with CUMS group.

According to our previous study (Huang et al., 2022), probiotic could decrease concentration of inflammatory cytokines IL-1β and IL-18, which were produced after NLRP3 inflammasome activation. Therefore, expression of NLRP3 inflammasome were measured by western blot and immunofluorescence. Results of western blot showed that CUMS stimulation increased the expression of NLRP3, Caspase-1 and ASC in brain and colon compared to Control group (*p<0.05)*. Probiotic intervention decreased the expression of NLRP3, Caspase-1 and ASC, in contrast to CUMS group (*p<0.05*, **Figures 2A-D** *)*. These results were also confirmed by immunofluorescence analysis. Compared with control group, positive cells of NLRP3, Caspase-1 and ASC in hippocampus and cortex were significantly increased. Administration with probiotic significantly reversed the effects of CUMS on NLRP3 inflammasome (*p<0.05*, **Figures 2E-H** *)*.

### 3.2 CUMS decreased the expression of tight junction proteins

**Figure 3:**
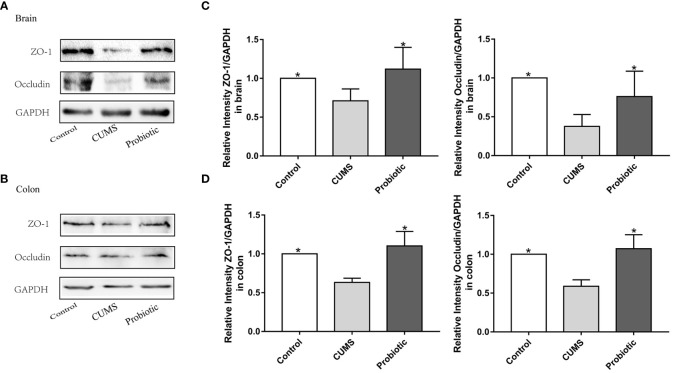
CUMS decreased the expression of tight junction proteins Occludin, ZO-1 in brain and colon, but probiotic alleviated the impairment of barrier function. Western blot was used to detect the expression of Occludin and ZO-1 in brain **(A)** and in colon **(B)**. **(C, D)** Immunoblot analysis for the protein levels of Occludin and ZO-1 in brain and colon. All data are expressed as the mean ± SD (n=5). *p<0.05, compared with CUMS group.

To further investigate the integrity of the barrier structures, the expression of Occludin and ZO-1, essential tight junction proteins of the gut barrier and blood-brain barrier, was measured by Western blot. As shown by the western blot results in **Figure 3A-D**, expression levels of Occludin and ZO-1 in brain and colon of CUMS group were significantly decreased compared with control group (*p*<0.05). Probiotic treatment significantly increased these proteins expression (*p*<0.05). These results suggested that probiotic supplementation ameliorated damaged barrier structures induced by CUMS stimulation.

### 3.3 Fecal transplantation from CUMS-exposed rat increased levels of IL-1β and IL-18 in the recipient rats

**Figure 4:**
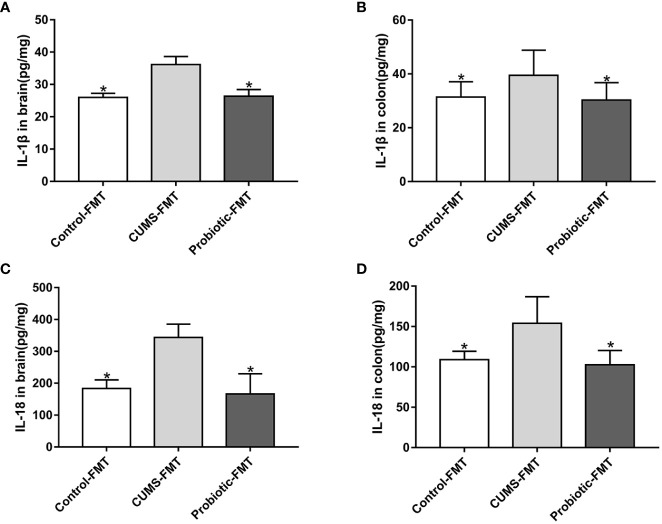
Concentrations of IL-1β and IL-18 in brain and colon of recipient rats. The levels of IL-1β in brain **(A)** and in colon **(B)**. The levels of IL-18 in brain **(C)** and in colon **(D)**. All data are expressed as the mean ± SD (n=5 in brain, n=8 in colon). *p<0.05, compared with CUMS-FMT group.

Effects of fecal transplantation on the levels of inflammatory cytokines IL-1β and IL-18 was evaluated. Compared with Control-FMT group, concentrations of IL-1β and IL-18 were increased in CUMS-FMT group(*p<0.05)*, which indicated that altered microbiota induced by CUMS stimulation increased the inflammation level in colon and brain. In addition, concentrations of IL-1β and IL-18 were decreased in the Probiotic-FMT group compared with CUMS-FMT group (*p<0.05*, **Figures 4A-D** *).*

### 3.4 Fecal transplantation from CUMS-exposed rat increased NLRP3 inflammasome expression in the recipient rats

**Figure 5:**
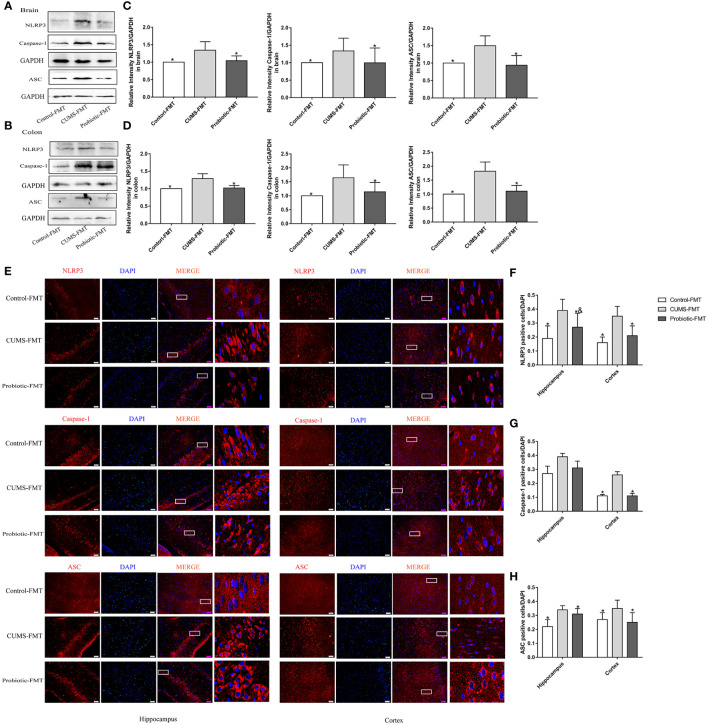
Fecal transplantation from CUMS-exposed rat increased the levels of NLRP3 inflammasome in brain and colon of recipient rats. **(A, B)** Representative western blot for NLRP3 inflammasome. **(C, D)** Bar graphs show semiquantitative levels of NLRP3 inflammasome as determined by band analysis. **(E)** Representative images of immunofluorescence staining for NLRP3 inflammasome in hippocampus and cortex. Scale bar=50 μm. **(F–H)** Quantification of positive cells expressing NLRP3 inflammasome/DAPI graphed. All data are expressed as the mean ± SD (n=5 in western blot, n=3 in immunofluorescence). *p<0.05, compared with CUMS-FMT group; ^&^ *p*<0.05, compared with Control-FMT group.

Expression levels of NLRP3, Caspase-1 and ASC in recipient rats were detected by western blot and immunofluorescence to determine the effect of microbiota on NLRP3 inflammasome activation. Results of western blot and immunofluorescence showed that NLRP3, Caspase-1 and ASC levels were upregulated in the CUMS-FMT group compared that in the Control-FMT group (*p<0.05)*. However, compared with CUMS-FMT group, levels of NLRP3, Caspase-1 and ASC in Probiotic-FMT group were lower (*p<0.05*, **Figures 5A-H** *)*. These results suggested that microbiota could influence NLRP3 inflammasome activation both in brain and colon.

### 3.5 Fecal transplantation from CUMS-exposed rat decreased tight junction proteins expression in the recipient rats

**Figure 6:**
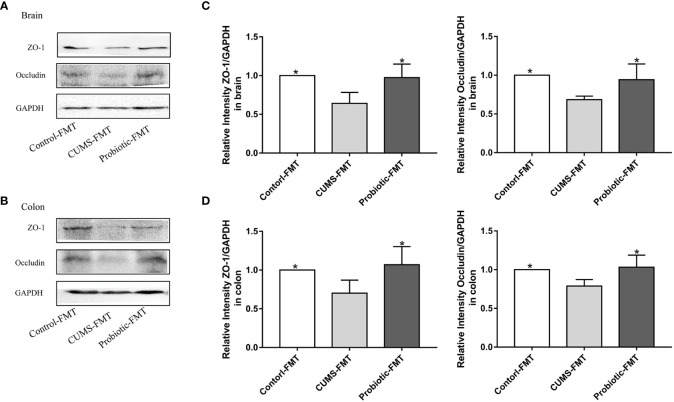
Fecal transplantation from CUMS-exposed rat decreased the levels of tight junction proteins in brain and colon of recipient rats. **(A, B)** Representative western blot for Occludin and ZO-1 in brain and colon, respectively. **(C, D)** Quantitative analysis of levels of Occludin and ZO-1 in brain and colon, respectively. All data are expressed as the mean ± SD (n=5). *p<0.05, compared with CUMS group.

Tight junction proteins Occludin and ZO-1 were measured to further explore the effects of microbiota on barrier function. Quantitative analysis revealed that fecal transplantation from CUMS rats exhibited the lower level of Occludin and ZO-1, compared with the Control-FMT group (*p<0.05)*. In addition, these tight junction proteins levels were significantly increased in the Probiotic-FMT group, compared with that in the CUMS-FMT group (*p<0.05*, **Figures 6A-D** ).

### 3.6 Fecal transplantation from CUMS-exposed rat intestinal altered microbiota in the recipient rats

**Figure 7:**
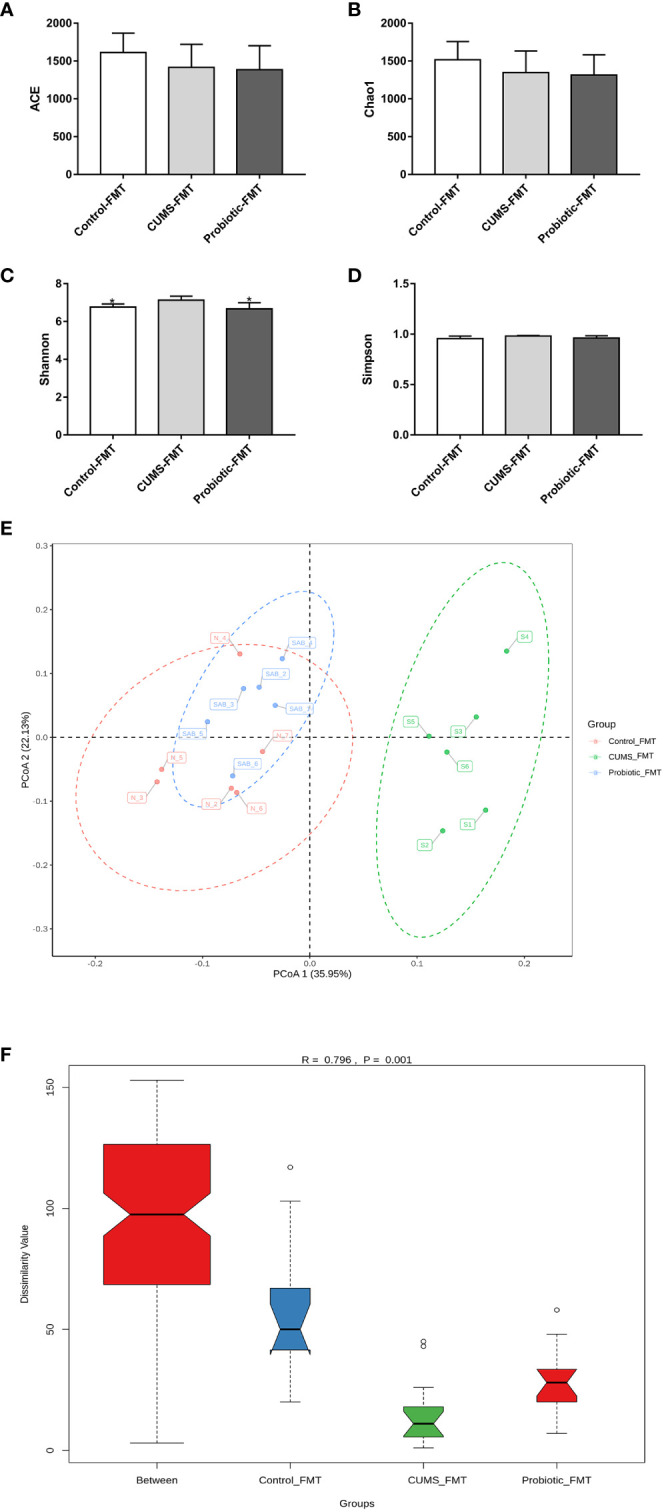
Effect of fecal transplantation from donor rats on microbiota Alpha and beta diversity in the recipient rats. **(A)** ACE index. **(B)** Chao1 index. **(C)** Shannon index. **(D)** Simpson index. **(E)** Unweighted PCoA analysis. **(F)** ANOSIM analysis. All data are expressed as the mean ± SD (n=6). *p<0.05, compared with CUMS group.

As shown in **Figures 7A-D**, ACE index, Simpson index and Chao1 index that reflected microbiota richness, exhibited lower levels in Probiotic-FMT group compared to CUMS-FMT group, but there was no significant difference (*p>0.05)*. However, compared with CUMS-FMT group, the Shannon index was significantly decreased in the Probiotic-FMT group (*p<0.05)*.

The unweighted PCoA analysis and ANOSIM analysis were performed to compare the beta diversity of microbiota among three groups. The results of PCoA analysis showed that the composition of microbiota in CUMS-FMT group was significantly separated from other groups, however the Probiotic-FMT group was close to Control-FMT group. Furthermore, ANOSIM analysis also supported that a significant difference in microbiota community structure between three groups (*p<0.05*, **Figures 7E-F** *)*.

**Figure 8:**
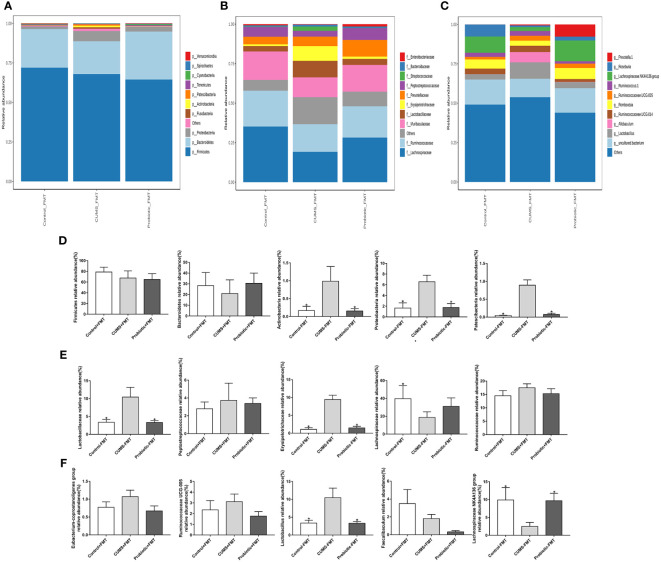
Effect of fecal transplantation from donor rats on relative abundances of some species in the recipient rats. **(A–C)** Heat map of microbiota in phylum, family and genus. **(D)** Comparison of relative abundances of microbiota at the phylum level in the three groups. **(E)** Comparison of relative abundances of microbiota at the family level in the three groups. **(F)** Comparison of relative abundances of microbiota at the genus level in the three groups. All data are expressed as the mean ± SD (n=6). *p<0.05, compared with CUMS group.

The heat map of microbiota in phylum, family and genus as shown in **Figures 8A-C**. Composition of microbiota at phylum level was significant differences among the three groups. The **Figure 8D** revealed the composition of phylum, and *firmicutes*, *facteroidetes*, *actinobacteria*, *proteobacteria* and *patescibacteria* were the dominant microbiota. The relative abundance of *actinobacteria*, *proteobacteria* and *patescibacteria* were significantly higher in the CUMS-FMT group than those in the Control-FMT group (*p<0.05)*, while the relative abundance of these microbiota was significantly downregulated in the Probiotic-FMT group relative to the CUMS-FMT group (*p<0.05)*. There was no significant difference in the relative abundance of *firmicutes* and *bacteroidetes* between the three groups (*p>0.05)*, but the abundance of *bacteroidetes* in the Probiotic-FMT group was superior to that in the CUMS-FMT group.

At the family level, the relative abundance of five primary microbiotas were analyzed (**Figure 8E**). Compared with Control-FMT group, the relative abundance of *lactobacillaceae* and *erysipelotrichichaceae* were obviously upregulated in the CUMS-FMT group, but the relative abundance of *lachnospiraceae* was significantly downregulated (*p<0.05)*. Furthermore, fecal transplantation from probiotic treatment rats showed a higher abundance of *lactobacillaceae* and *erysipelotrichichaceae* compared to the CUMS-FMT group (*p<0.05)*. Meanwhile, compared with CUMS-FMT group, the relative abundance of *peptostreptocollaceae* and *ruminococcaceae* were downregulated in the Probiotic-FMT group, but the relative abundance of *lachnospiraceae* was upregulated, while no significant difference was found (*p>0.05)*.

Similarly, the relative abundance of genus *lactobacillus* in the CUMS-FMT group was significantly increased relative to that in the Control-FMT group (*p<0.05)*, but the relative abundance of *lachnospiraceae NK4A136 group*, which belonged to family *lachnospiraceae* was significantly decreased (*p<0.05)*. Importantly, the relative abundance of *lactobacillus* and *lachnospiraceae NK4A136 group* in the Probiotic-FMT group were significantly decreased and increased respectively compared to the CUMS-FMT group (*p<0.05)*. Probiotic-FMT group showed lower relative abundance of *eubacterium-coprostanoligenes group*, *ruminococcaceae UCG-005* and *faecalibaculum* compared with CUMS-FMT group, but there was no significant difference (**Figure 8F**, *p>0.05)*. In conclusion, these results suggested that the composition of microbiota in the recipient rat were partially consistent with that of the donor rats (Huang et al., 2022).

## 4 Discussion

Both clinical and animal studies indicated that microbiota could influence depression by regulating hormonal, metabolic and immune *via* gut-brain axis (Foster and McVey Neufeld, 2013). In this study, results indicated that CUMS stimulation increased the levels of NLRP3, Caspase-1 and ASC, while probiotic, a common method of changing microbiota, suppressed the expression of the NLRP3 inflammasome. Importantly, fecal transplantation from CUMS-exposed rat induced the activation of NLRP3 inflammasome. Meanwhile, alterations of tight junction proteins and microbiota in recipient rats were partially consistent with those in donor rats. These results suggested that microbiota was associated with NLRP3 inflammasome.

Fecal transplantation, as a microbiota-targeted technique, could significantly change gut microbial community in recipient *via* delivering infusion feces (the entire gut microbiota) of donor (Khoruts and Weingarden, 2014). In our study, the diversity and richness of intestinal flora in recipient rats, as reflected by ACE index, Chao1 index and Simpson index, and five dominant microbiotas in terms of phylum, family and genus were consistent with that of the donor rats (Huang et al., 2022), which further indicated that microbiota of recipient rats were influenced by the microbiota of donor rats. Consistent with other studies (Yu et al., 2017; An et al., 2020), the relative abundance of *actinobacteria*, *proteobacteria* and *patescibacteria* increased, while the abundance of *bacteroidetes* decreased in recipient rats that received fecal liquid from CUMS rats, suggested that chronic stress could alter the composition of gut microbiota (Galley et al., 2014; Marin et al., 2017). However, there were some disparities in microbiota between recipient and donor rats, such as upregulated relative abundance of *firmicutes* in CUMS donor rats, but downregulated relative abundance of *firmicutes* in CUMS recipient rats, and the alteration of Shannon index. Due to antibiotic intervention could deplete microbiota and affect the colonization of the intestinal flora, thus the microbiota of recipient rats was failed to completely overlap with that of the donor rats (Ceylani et al., 2018). In addition, microbiota structure may be influenced by many factors, such as genetic, age, source of the rats, breeding environment, etc (Laukens et al., 2016). Interestingly, these alterations of microbiota in Probiotic-FMT group were reversed, consistent with probiotic donor rats. Our previous study has shown that combined *lactobacillus rhamnosus* and *bifidobacterium* could alleviate depression-like behaviors induced by CUMS stress (Huang et al., 2022). These results suggested that gut microbial dysbiosis could potentially contribute to the development and manifestation of depression, and that probiotics may be effective in altering the microbiota and alleviating depression.

Inflammation was an important cause of depression, and increased inflammation cytokines was related to depression, particularly IL-1β (Yirmiya et al., 2015). NLRP3 inflammasome activated by a variety of pathogen-associated molecular patterns or damage-associated molecular patterns could promote IL-1β production (Gurung et al., 2015; Broz and Dixit, 2016). Increasing researches demonstrated that NLRP3 activation was the key factor in the pathogenesis of depression (Zhang et al., 2014; Zhang et al., 2015; Kim et al., 2016). Meanwhile, there was a certain association between gut microbiota and NLRP3 inflammasome. For example, activated NLRP3 inflammasome by microbiota affects the acute pancreatitis (Li et al., 2020), and the expression of NLRP3 also shapes the composition of the intestinal flora (Zhang et al., 2019), but the detailed understanding of their interactions in depression is lacking. In this study, our results indicated that levels of NLRP3, Caspase-1 and ASC were increased in the brain of CUMS rats, as found in other studies (Wang et al., 2021; Xie et al., 2021). Similarly, elevated NLRP3 inflammasome and IL-1β and IL-18 also were found in recipient rats gavaged fecal solution from CUMS donor rats, which suggested that altered microbiota induced by CUMS stimulus affected activation of NLRP3 inflammasome. Chronic stress was the risk factors and etiology for several gastrointestinal diseases such as functional intestinal disorders and inflammatory bowel diseases (IBDs), and chronic stress also increased the risk of ulcerative colitis (UC) recurrence (Levenstein et al., 2000; Ringel and Drossman, 2001). Moreover, depression patients and animal also might suffer from IBD (Yanartas et al., 2016; Wei et al., 2019). Our results found that CUMS treatment increased the expression of NLRP3 inflammasome and inflammatory cytokines in colon, which further indicated that depression and IBD might co-occur. In addition, downregulated levels of NLRP3 inflammasome were found in both probiotic donor and recipient rats, which suggested that probiotic treatment could reduce inflammation caused by CUMS stimulation *via* altering the intestinal flora. Our previous study has shown that combined probiotic could improve microbiota and depression-like behaviors. Therefore, probiotics may improve depression by altering the intestinal flora and inhibiting the activation of NLRP3 inflammasomes.

The tight junction proteins Occludin and ZO-1 formed the structures of intestinal mucosal barrier and blood-brain barrier (Li et al., 2018; Kealy et al., 2020). Studies found that stress can break the intestinal mucosal barrier and increase intestinal permeability, leading to the entry of bacterial metabolites and endotoxins into periphery or brain, which elevated inflammation (Söderholm et al., 2002; Vanuytsel et al., 2014). In fact, some neuropsychiatric disorders, such as Parkinson’s disease, Alzheimer’ disease and depression, have been linked to leaky gut and destruction of the blood-brain barrier (Pellegrini et al., 2018; Tikiyani and Babu, 2019). Therefore, the expression of tight junction proteins and the adequate function of the intestinal barrier were essential to prevent the development of associated neurological disorders, including depression. In this study, our results found that the expression of Occludin and ZO-1 was decreased in CUMS donor and recipient rats, indicating that microbiota could damage the barrier function by downregulating expression of tight junction proteins, thus decreasing the intestinal mucosal barrier and blood-brain barrier, thus improving the harmful components accessed to periphery, thus increasing inflammation (Rhee et al., 2009; Maqsood and Stone, 2016). Meanwhile, increased levels of Occludin and ZO-1 were found in probiotic donor and recipient rats, suggesting that probiotic could decrease inflammation *via* amending barrier function.

## 5 Conclusion

In this study, we found that the alteration microbiota induced by chronic stress activated the NLRP3 inflammasome and then increased the inflammatory cytokines in brain, leading to depressive-like behaviors. Furthermore, probiotic could improve depression-like behaviors by amending the microbiota and suppressing the activation of NLRP3 inflammasome. This study revealed a new mechanism of CUMS-induced depressive behaviors and provided a new therapeutic direction for prevention and treatment of depression.

## 6 Limitations of the current study

The absence of antibiotics treatment group makes it hard to exclude possible effect of antibiotics on microbiota. In addition, the recipient rats were not experienced CUMS stimulation and behavioral test, the relationship between intestinal flora and depression-like behavior lack direct evidence. Thirdly, we measured only the tight junction proteins Occludin and ZO-1 to reflect the permeability of the intestinal barrier and blood-brain barrier, which were only part of gut-brain axis. Finally, the sample size of the study was limited and solely concentrated on the impact of probiotic intervention for a 30-day period, rather than examining the effects of prolonged probiotic intervention. Therefore, further studies with larger samples and longer follow-up periods are needed to explore the effect of probiotic on depression-like behaviors *via* gut-brain axis.

## Data availability statement

The dataset presented in this study can be found in the online repository. The name and accession number of the repository/repository can be found below: https://www.ncbi.nlm.nih.gov/bioproject/PRJNA951082, PRJNA951082.

## Ethics statement

The animal study was reviewed and approved by Animal Ethical and Welfare Committee of Tianjin Nankai Hospital.

## Author contributions

The author’s responsibility were as follows - HL and XLZ designed the research (project conception, development of overall research plan, and study oversight); LH, ZM conducted the research (conduct of the experiment and data collection); XRZ, MZ, XL and YZ analyzed the data or performed the statistical analysis; LH and HL wrote the manuscript; and HL had primary responsibility for the final content. LH, ZM and XLZ contributed equally to the work. All authors contributed to the article and approved the submitted version.

## References

[B1] Alcocer-Gómez E. de Miguel M. Casas-Barquero N. Núñez-Vasco J. Sánchez-Alcazar J. A. Fernández-Rodríguez A.. (2014). NLRP3 inflammasome is activated in mononuclear blood cells from patients with major depressive disorder. Brain Behav. Immun. 36, 111–117. doi: 10.1016/j.bbi.2013.10.017 24513871

[B2] An Q. Li C. Chen Y. Yang Y. Song R. Zhou L.. (2020). Scaffold hopping of agomelatine leads to enhanced antidepressant effects by modulation of gut microbiota and host immune responses. Pharmacol. Biochem. Behav. 192, 172910. doi: 10.1016/j.pbb.2020.172910 32194087

[B3] Anderson G. Seo M. Berk M. Carvalho A. F. Maes M. (2016). Gut permeability and microbiota in parkinson’s disease: role of depression, tryptophan catabolites, oxidative and nitrosative stress and melatonergic pathways. Curr. Pharm. Des. 22 (40), 6142–6151. doi: 10.2174/1381612822666160906161513 27604608

[B4] Arioz B. I. Tastan B. Tarakcioglu E. Tufekci K. U. Olcum M. Ersoy N.. (2019). Melatonin attenuates LPS-induced acute depressive-like behaviors and microglial NLRP3 inflammasome activation through the SIRT1/Nrf2 pathway. Front. Immunol. 10. doi: 10.3389/fimmu.2019.01511 PMC6615259 31327964

[B5] Broz P. Dixit V. M. (2016). Inflammasomes: mechanism of assembly, regulation and signalling. Nat. Rev. Immunol. 16 (7), 407–420. doi: 10.1038/nri.2016.58 27291964

[B6] Ceylani T. Jakubowska-Doğru E. Gurbanov R. Teker H. T. Gozen A. G. (2018). The effects of repeated antibiotic administration to juvenile BALB/c mice on the microbiota status and animal behavior at the adult age. Heliyon 4 (6), e00644. doi: 10.1016/j.heliyon.2018.e00644 29872772 PMC5986162

[B7] Desbonnet L. Clarke G. O’Sullivan O. Cotter P. D. Dinan T. G. Cryan J. F. (2015). Re: gut microbiota depletion from early adolescence in mice: implications for brain and behaviour. Brain Behav. Immun. 50, 335–336. doi: 10.1016/j.bbi.2015.07.011 26271708

[B8] Foster J. A. McVey Neufeld K. A. (2013). Gut-brain axis: how the microbiome influences anxiety and depression. Trends Neurosci. 36 (5), 305–312. doi: 10.1016/j.tins.2013.01.005 23384445

[B9] Fu Q. Zhai Z. Wang Y. Xu L. Jia P. Xia P.. (2018). NLRP3 deficiency alleviates severe acute pancreatitis and pancreatitis-associated lung injury in a mouse model. BioMed. Res. Int. 2018, 1294951. doi: 10.1155/2018/1294951 30622955 PMC6304199

[B10] Galley J. D. Nelson M. C. Yu Z. Dowd S. E. Walter J. Kumar P. S.. (2014). Exposure to a social stressor disrupts the community structure of the colonic mucosa-associated microbiota. BMC Microbiol. 14, 189. doi: 10.1186/1471-2180-14-189 25028050 PMC4105248

[B11] Guo H. Callaway J. B. Ting J. P. (2015). Inflammasomes: mechanism of action, role in disease, and therapeutics. Nat. Med. 21 (7), 677–687. doi: 10.1038/nm.3893 26121197 PMC4519035

[B12] Gurung P. Lukens J. R. Kanneganti T. D. (2015). Mitochondria: diversity in the regulation of the NLRP3 inflammasome. Trends Mol. Med. 21, 193–201. doi: 10.1016/j.molmed.2014.11.008 25500014 PMC4352396

[B13] Haneklaus M. O’Neill L. A. Coll R. C. (2013). Modulatory mechanisms controlling the NLRP3 inflammasome in inflammation: recent developments. Curr. Opin. Immunol. 25 (1), 40–45. doi: 10.1016/j.coi.2012.12.004 23305783

[B14] Hao W. Wu J. Yuan N. Gong L. Huang J. Ma Q.. (2021). Xiaoyaosan improves antibiotic-induced depressive-like and anxiety-like behavior in mice through modulating the gut microbiota and regulating the NLRP3 inflammasome in the colon. Front. Pharmacol. 12:619103. doi: 10.3389/fphar.2021.619103 33935710 PMC8087337

[B15] Heneka M. T. McManus R. M. Latz E. (2018). Inflammasome signalling in brain function and neurodegenerative disease. Nat. Rev. Neurosci. 19 (10), 610–621. doi: 10.1038/s41583-018-0055-7 30206330

[B16] Huang L. Lv X. Ze X. Ma Z. Zhang X. He R.. (2022). Combined probiotics attenuate chronic unpredictable mild stress-induced depressive-like and anxiety-like behaviors in rats. Front. Psychiatry 13. doi: 10.3389/fpsyt.2022.990465 PMC9490273 36159940

[B17] Jiang H. Ling Z. Zhang Y. Mao H. Ma Z. Yin Y.. (2015). Altered fecal microbiota composition in patients with major depressive disorder. Brain Behav. Immun. 48, 186–194. doi: 10.1016/j.bbi.2015.03.016 25882912

[B18] Julio-Pieper M. Bravo J. A. Aliaga E. Gotteland M. (2014). Review article: intestinal barrier dysfunction and central nervous system disorders–a controversial association. Aliment. Pharmacol. Ther. 40 (10), 1187–1201. doi: 10.1111/apt.12950 25262969

[B19] Kaufmann F. N. Costa A. P. Ghisleni G. Diaz A. P. Rodrigues A. L. S. Peluffo H.. (2017). NLRP3 inflammasome-driven pathways in depression: clinical and preclinical findings. Brain Behav. Immun. 64, 367–383. doi: 10.1016/j.bbi.2017.03.002 28263786

[B20] Kealy J. Greene C. Campbell M. (2020). Blood-brain barrier regulation in psychiatric disorders. Neurosci. Lett. 726, 133664. doi: 10.1016/j.neulet.2018.06.033 29966749

[B21] Khoruts A. Weingarden A. R. (2014). Emergence of fecal microbiota transplantation as an approach to repair disrupted microbial gut ecology. Immunol. Lett. 162 (2 Pt A), 77–81. doi: 10.1016/j.imlet.2014.07.016 25106113 PMC5554112

[B22] Kim H. K. Andreazza A. C. Elmi N. Chen W. Young L. T. (2016). Nod-like receptor pyrin containing 3 (NLRP3) in the post-mortem frontal cortex from patients with bipolar disorder: a potential mediator between mitochondria and immune-activation. J. Psychiatr. Res. 72, 43–50. doi: 10.1016/j.jpsychires.2015.10.015 26540403

[B23] Köhler O. Benros M. E. Nordentoft M. Farkouh M. E. Iyengar R. L. Mors O.. (2014). Effect of anti-inflammatory treatment on depression, depressive symptoms, and adverse effects: a systematic review and meta-analysis of randomized clinical trials. JAMA Psychiatry 71 (12), 1381–1391. doi: 10.1001/jamapsychiatry.2014.1611 25322082

[B24] Lai J. Zhang P. Jiang J. Mou T. Li Y. Xi C.. (2021). New evidence of gut microbiota involvement in the neuropathogenesis of bipolar depression by TRANK1 modulation: joint clinical and animal data. Front. Immunol. 12. doi: 10.3389/fimmu.2021.789647 PMC8724122 34992606

[B25] Laukens D. Brinkman B. M. Raes J. De Vos M. Vandenabeele P. (2016). Heterogeneity of the gut microbiome in mice: guidelines for optimizing experimental design. FEMS Microbiol. Rev. 40 (1), 117–132. doi: 10.1093/femsre/fuv036 26323480 PMC4703068

[B26] Levenstein S. Prantera C. Varvo V. Scribano M. L. Andreoli A. Luzi C.. (2000). Stress and exacerbation in ulcerative colitis: a prospective study of patients enrolled in remission. Am. J. Gastroenterol. 95 (5), 1213–1220. doi: 10.1111/j.1572-0241.2000.02012 10811330

[B27] Li C. Cai Y. Y. Yan Z. X. (2018). Brain-derived neurotrophic factor preserves intestinal mucosal barrier function and alters gut microbiota in mice. Kaohsiung. J. Med. Sci. 34 (3), 134–141. doi: 10.1016/j.kjms.2017.11.002 29475460 PMC11915681

[B28] Li X. He C. Li N. Ding L. Chen H. Wan J.. (2020). The interplay between the gut microbiota and NLRP3 activation affects the severity of acute pancreatitis in mice. Gut Microbes 11 (6), 1774–1789. doi: 10.1080/19490976.2020.1770042 32529941 PMC7524163

[B29] Li Y. Song W. Tong Y. Zhang X. Zhao J. Gao X.. (2021). Isoliquiritin ameliorates depression by suppressing NLRP3-mediated pyroptosis *via* miRNA-27a/SYK/NF-κB axis. J. Neuroinflamm. 18 (1), 1. doi: 10.1186/s12974-020-02040-8 PMC7786465 33402173

[B30] Li N. Wang Q. Wang Y. Sun A. Lin Y. Jin Y.. (2019). Fecal microbiota transplantation from chronic unpredictable mild stress mice donors affects anxiety-like and depression-like behavior in recipient mice *via* the gut microbiota-inflammation-brain axis. Stress 22 (5), 592–602. doi: 10.1080/10253890.2019.1617267 31124390

[B31] Lowe P. P. Gyongyosi B. Satishchandran A. Iracheta-Vellve A. Cho Y. Ambade A.. (2018). Reduced gut microbiome protects from alcohol-induced neuroinflammation and alters intestinal and brain inflammasome expression. J. Neuroinflamm. 15 (1), 298. doi: 10.1186/s12974-018-1328-9 PMC6203993 30368255

[B32] Maqsood R. Stone T. W. (2016). The gut-brain axis, BDNF, NMDA and CNS disorders. Neurochem. Res. 41 (11), 2819–2835. doi: 10.1007/s11064-016-2039-1 27553784

[B33] Marin I. A. Goertz J. E. Ren T. Rich S. S. Onengut-Gumuscu S. Farber E.. (2017). Microbiota alteration is associated with the development of stress-induced despair behavior. Sci. Rep. 7, 43859. doi: 10.1038/srep43859 28266612 PMC5339726

[B34] Miao E. A. Rajan J. V. Aderem A. (2011). Caspase-1-induced pyroptotic cell death. Immunol. Rev. 243, 206–214. doi: 10.1111/j.1600-065X.2011.01044.x 21884178 PMC3609431

[B35] Mou Y. Du Y. Zhou L. Yue J. Hu X. Liu Y.. (2022). Gut microbiota interact with the brain through systemic chronic inflammation: implications on neuroinflammation, neurodegeneration, and aging. Front. Immunol. 13. doi: 10.3389/fimmu.2022.796288 PMC9021448 35464431

[B36] Pan Y. Chen X. Y. Zhang Q. Y. Kong L. D. (2014). Microglial NLRP3 inflammasome activation mediates IL-1β-related inflammation in prefrontal cortex of depressive rats. Brain Behav. Immun. 41, 90–100. doi: 10.1016/j.bbi.2014.04.007 24859041

[B37] Pellegrini C. Antonioli L. Colucci R. Blandizzi C. Fornai M. (2018). Interplay among gut microbiota, intestinal mucosal barrier and enteric neuro-immune system: a common path to neurodegenerative diseases? Acta Neuropathol. 136 (3), 345–361. doi: 10.1007/s00401-018-1856-5 29797112

[B38] Perry B. I. Upthegrove R. Kappelmann N. Jones P. B. Burgess S. Khandaker G. M. (2021). Associations of immunological proteins/traits with schizophrenia, major depression and bipolar disorder: a bi-directional two-sample mendelian randomization study. Brain Behav. Immun. 97, 176–185. doi: 10.1016/j.bbi.2021.07.009 34280516 PMC7612947

[B39] Rhee S. H. Pothoulakis C. Mayer E. A. (2009). Principles and clinical implications of the brain-gut-enteric microbiota axis. Nat. Rev. Gastroenterol. Hepatol. 6 (5), 306–314. doi: 10.1038/nrgastro.2009.35 19404271 PMC3817714

[B40] Ringel Y. Drossman D. A. (2001). Psychosocial aspects of crohn’s disease. Surg. Clin. North Am. 81 (1), 231–52, x. doi: 10.1016/s0039-6109(05)70283-8 11218167

[B41] Sarkar A. Lehto S. M. Harty S. Dinan T. G. Cryan J. F. Burnet P. W. J. (2016). Psychobiotics and the manipulation of bacteria-Gut-Brain signals. Trends Neurosci. 39 (11), 763–781. doi: 10.1016/j.tins.2016.09.002 27793434 PMC5102282

[B42] Söderholm J. D. Yates D. A. Gareau M. G. Yang P. C. MacQueen G. Perdue M. H. (2002). Neonatal maternal separation predisposes adult rats to colonic barrier dysfunction in response to mild stress. Am. J. Physiol. Gastrointest. Liver. Physiol. 283 (6), G1257–G1263. doi: 10.1152/ajpgi.00314.2002 12388189

[B43] Su W. J. Zhang Y. Chen Y. Gong H. Lian Y. J. Peng W.. (2017). NLRP3 gene knockout blocks NF-κB and MAPK signaling pathway in CUMS-induced depression mouse model. Behav. Brain Res. 322 (Pt A), 1–8. doi: 10.1016/j.bbr.2017.01.018 28093255

[B44] Syed S. A. Beurel E. Loewenstein D. A. Lowell J. A. Craighead W. E. Dunlop B. W.. (2018). Defective inflammatory pathways in never-treated depressed patients are associated with poor treatment response. Neuron 99 (5), 914–924. doi: 10.1016/j.neuron.2018.08.001 30146307 PMC6151182

[B45] Tikiyani V. Babu K. (2019). Claudins in the brain: unconventional functions in neurons. Traffic 20 (11), 807–814. doi: 10.1111/tra.12685 31418988

[B46] Vanuytsel T. van Wanrooy S. Vanheel H. Vanormelingen C. Verschueren S. Houben E.. (2014). Psychological stress and corticotropin-releasing hormone increase intestinal permeability in humans by a mast cell-dependent mechanism. Gut 63 (8), 1293–1299. doi: 10.1136/gutjnl-2013-305690 24153250

[B47] Wang Y. L. Wu H. R. Zhang S. S. Xiao H. L. Yu J. Ma Y. Y.. (2021). Catalpol ameliorates depressive-like behaviors in CUMS mice *via* oxidative stress-mediated NLRP3 inflammasome and neuroinflammation. Transl. Psychiatry 11 (1), 353. doi: 10.1038/s41398-021-01468-7 34103482 PMC8187638

[B48] Wei L. Li Y. Tang W. Sun Q. Chen L. Wang X.. (2019). Chronic unpredictable mild stress in rats induces colonic inflammation. Front. Physiol. 10. doi: 10.3389/fphys.2019.01228 PMC6764080 31616319

[B49] Wong M. L. Inserra A. Lewis M. D. Mastronardi C. A. Leong L. Choo J.. (2016). Inflammasome signaling affects anxiety- and depressive-like behavior and gut microbiome composition. Mol. Psychiatry 21, 797–805. doi: 10.1038/mp.2016.46 27090302 PMC4879188

[B50] Xie J. Bi B. Qin Y. Dong W. Zhong J. Li M.. (2021). Inhibition of phosphodiesterase-4 suppresses HMGB1/RAGE signaling pathway and NLRP3 inflammasome activation in mice exposed to chronic unpredictable mild stress. Brain Behav. Immun. 92, 67–77. doi: 10.1016/j.bbi.2020.11.029 33221489

[B51] Yan T. Nian T. Liao Z. Xiao F. Wu B. Bi K.. (2020). Antidepressant effects of a polysaccharide from okra (Abelmoschus esculentus (L) moench) by anti-inflammation and rebalancing the gut microbiota. Int. J. Biol. Macromol. 144, 427–440. doi: 10.1016/j.ijbiomac.2019.12.138 31862370

[B52] Yanartas O. Kani H. T. Bicakci E. Kilic I. Banzragch M. Acikel C.. (2016). The effects of psychiatric treatment on depression, anxiety, quality of life, and sexual dysfunction in patients with inflammatory bowel disease. Neuropsychiatr. Dis. Treat. 12, 673–683. doi: 10.2147/NDT.S106039 27069364 PMC4818049

[B53] Yang Y. Xing M. J. Li Y. Zhang H. F. Yuan T. F. Peng D. H. (2021). Reduced NLRP3 inflammasome expression in the brain is associated with stress resilience. Psychoneuroendocrinology 128, 105211. doi: 10.1016/j.psyneuen.2021.105211 33812228

[B54] Yirmiya R. Rimmerman N. Reshef R. (2015). Depression as a microglial disease. Trends Neurosci. 38, 637–658. doi: 10.1016/j.tins.2015.08.001 26442697

[B55] Yu M. Jia H. Zhou C. Yang Y. Zhao Y. Yang M.. (2017). Variations in gut microbiota and fecal metabolic phenotype associated with depression by 16S rRNA gene sequencing and LC/MS-based metabolomics. J. Pharm. BioMed. Anal. 138, 231–239. doi: 10.1016/j.jpba.2017.02.008 28219800

[B56] Zhang Y. Huang R. Cheng M. Wang L. Chao J. Li J.. (2019). Gut microbiota from NLRP3-deficient mice ameliorates depressive-like behaviors by regulating astrocyte dysfunction *via* circHIPK2. Microbiome 7 (1), 116. doi: 10.1186/s40168-019-0733-3 31439031 PMC6706943

[B57] Zhang Y. Liu L. Liu Y. Z. Shen X. L. Wu T. Y. Zhang T.. (2015). NLRP3 inflammasome mediates chronic mild stress-induced depression in mice via neuroinflammation. Int. J. Neuropsychopharmacol. 18 (8), pyv006. doi: 10.1093/ijnp/pyv006 25603858 PMC4571628

[B58] Zhang Y. Liu L. Peng Y. L. Liu Y. Z. Wu T. Y. Shen X. L.. (2014). Involvement of inflammasome activation in lipopolysaccharide-induced mice depressive-like behaviors. CNS Neurosci. Ther. 20 (2), 119–124. doi: 10.1111/cns.12170 24279434 PMC6493120

[B59] Zhen Y. Zhang H. (2019). NLRP3 inflammasome and inflammatory bowel disease. Front. Immunol. 10. doi: 10.3389/fimmu.2019.00276 PMC6403142 30873162

[B60] Zheng P. Zeng B. Zhou C. Liu M. Fang Z. Xu X.. (2016). Gut microbiome remodeling induces depressive-like behaviors through a pathway mediated by the host’s metabolism. Mol. Psychiatry 21 (6), 786–796. doi: 10.1038/mp.2016.44 27067014

